# Geography and genography: prediction of continental origin using randomly selected single nucleotide polymorphisms

**DOI:** 10.1186/1471-2164-8-68

**Published:** 2007-03-10

**Authors:** Dominic J Allocco, Qing Song, Gary H Gibbons, Marco F Ramoni, Isaac S Kohane

**Affiliations:** 1 Children's Hospital Informatics Program at Harvard-MIT Division of Health Sciences and Technology, Boston, MA, USA; 2 Division of Cardiology, Beth Israel Deaconess Medical Center, Boston, MA, USA; 3 Cardiovascular Research Institute, Morehouse School of Medicine, Atlanta, GA, USA; 4 Harvard Partners Center for Genetics and Genomics, Boston, MA, USA

## Abstract

**Background:**

Recent studies have shown that when individuals are grouped on the basis of genetic similarity, group membership corresponds closely to continental origin. There has been considerable debate about the implications of these findings in the context of larger debates about race and the extent of genetic variation between groups. Some have argued that clustering according to continental origin demonstrates the existence of significant genetic differences between groups and that these differences may have important implications for differences in health and disease. Others argue that clustering according to continental origin requires the use of large amounts of genetic data or specifically chosen markers and is indicative only of very subtle genetic differences that are unlikely to have biomedical significance.

**Results:**

We used small numbers of randomly selected single nucleotide polymorphisms (SNPs) from the International HapMap Project to train naïve Bayes classifiers for prediction of ancestral continent of origin. Predictive accuracy was tested on two independent data sets. Genetically similar groups should be difficult to distinguish, especially if only a small number of genetic markers are used. The genetic differences between continentally defined groups are sufficiently large that one can accurately predict ancestral continent of origin using only a minute, randomly selected fraction of the genetic variation present in the human genome. Genotype data from only 50 random SNPs was sufficient to predict ancestral continent of origin in our primary test data set with an average accuracy of 95%. Genetic variations informative about ancestry were common and widely distributed throughout the genome.

**Conclusion:**

Accurate characterization of ancestry is possible using small numbers of randomly selected SNPs. The results presented here show how investigators conducting genetic association studies can use small numbers of arbitrarily chosen SNPs to identify stratification in study subjects and avoid false positive genotype-phenotype associations. Our findings also demonstrate the extent of variation between continentally defined groups and argue strongly against the contention that genetic differences between groups are too small to have biomedical significance.

## Background

A major goal of both the Human Genome Project and the subsequent International HapMap Project was to provide a foundation for understanding genetic variation in the human genome [1,2]. Multiple studies over the past several decades have consistently concluded that only 5–15% of human genetic variation can be explained by differences between populations [3-6]. Although this proportion is relatively small, recent studies have shown that when individuals are grouped on the basis of genetic similarity, group membership corresponds closely to ancestral geographic origin [5] or self-identified race or ethnicity [7].

There has been considerable discussion about the implications of these findings in the context of larger debates about population sub-structure, race and genetics [8-13]. One major aspect of the debate concerns the extent of genetic variation between groups. This debate is becoming less theoretical as targeted therapies like BiDil (a branded combination of hydralazine and isosorbide dinitrate approved by the FDA for the treatment of congestive heart failure in African-Americans) are introduced. Some have argued that the genetic differences between continentally defined groups are relatively small and thus unlikely to have biomedical significance [3,14]. Providing support for this view, others have noted that accurate classification of ancestral origin, while possible, requires large numbers of genetic markers [9-12,15]. The opposing viewpoint holds that there are significant genetic differences between groups and that these genetic differences may account for differences in risk of disease between populations [16]. Proponents of this viewpoint also note that an understanding of genetic variation between groups is important to avoid confounding in genotype-phenotype association studies [7,17].

A variety of different computational methods, including those implemented in the popular programs STRUCTURE and BAPS, have been developed and used to explore genetic variation in populations [18-20]. Previous investigators have used variation in both mitochondrial DNA and microsatellites to characterize population sub-structure and ancestral geographic origin [5,7,21-23]. Analysis of the relationship between genetic variation in chromosomal (as opposed to mitochondrial) SNPs and ancestral geographic origin has been more limited because large scale, genome-wide SNP data from geographically diverse individuals has not been available. Lao et al recently used data from Affymetrix 10K arrays to identify 10 specific SNPs that were highly informative for characterizing ancestry. These SNPs were found to be somewhat less informative when used to characterize an independent data set.

In this paper, we use recently published genome-wide SNP data to analyze population sub-structure and ancestral geographic origin. The analysis uses a much larger number of SNPs than has previously been possible and focuses on the use of randomly selected SNPs. We show that one can accurately predict continent of origin in independent data sets using only a very small number of randomly selected SNPs. Genotype data from 50 random SNPs is sufficient to predict ancestral continent of origin in our primary test data set with an average accuracy of 95%. We analyze the comparative utility of SNPs in introns, coding exons, regulatory regions and regions coding for untranslated mRNA for prediction of ancestry. Finally, we show that SNPs that are informative about ancestry are common and widely distributed throughout the genome. Our findings demonstrate how researchers conducting SNP based genotype-phenotype association studies can accurately and reproducibly characterize ancestry using random SNPs, as opposed to SNPs specifically chosen to be informative about ancestry. As genetically similar groups should be difficult to distinguish using only a small number of genetic markers, our results also demonstrate the extent of genetic variation between continentally defined groups and argue strongly against the contention that differences between groups are too small to have biomedical significance.

## Results

The International HapMap Project is a large collaborative effort that has made publicly available genotype data for 270 individuals from four different populations: Yoruba in Ibadan, Nigeria (YRI); Japanese in Tokyo, Japan (JPT); Han Chinese in Beijing (CHB), China; and Utah residents with ancestry from northern and western Europe (CEU) [2]. At the time of our study, genotype data that had passed quality control filters was available for almost 4 million SNPs. We used the HapMap data to develop classifiers for predicting ancestral continent of origin and tested these classifiers on independent data sets.

Our primary test data set consisted of 1,586,383 SNPs genotyped by investigators at Perlegen Sciences to study DNA variation in human populations [24]. We excluded nine individuals of European ancestry in this data because they were also genotyped in the HapMap Project. Thus, this test data set included genotype data for 23 African-Americans, 15 European-Americans and 24 Han Chinese. The second test data set consisted of 4,124 SNPs genotyped as part of the Innate Immunity Program for Genomic Applications (IIPGA) and made publicly available on their website [25]. Nine individuals in this data set were also excluded from the analysis because they were genotyped in the HapMap Project. This test data set therefore included data for 24 African-Americans and 14 European-Americans. SNPs in the HapMap and Perlegen data sets were selected so as to be generally representative of variation throughout the genome, while SNPs in the IIPGA data set were selected by the original investigators for genotyping on the basis of potential involvement in the innate immune response.

For each of the test data sets, we limited our analysis to the bi-allelic SNPs that were present on autosomal chromosomes in both the HapMap data and the test set data. There were 1,047,543 and 1,588 such SNPs in the intersection of the HapMap data with the Perlegen and IIPGA data, respectively.

**Figure 1:**
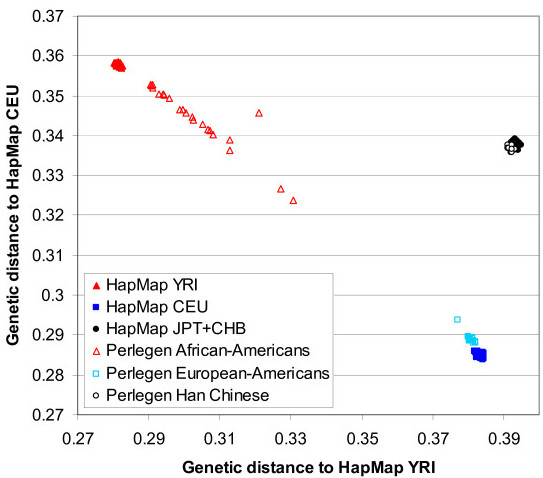
**Genetic distance from individuals in the Perlegen data to the three continentally defined HapMap groups**. Individuals of similar geographic origin cluster together. African-Americans from the Perlegen data set lie in between the HapMap Yoruba and European-Americans, but are much closer to the Yoruba.

**Figure 2:**
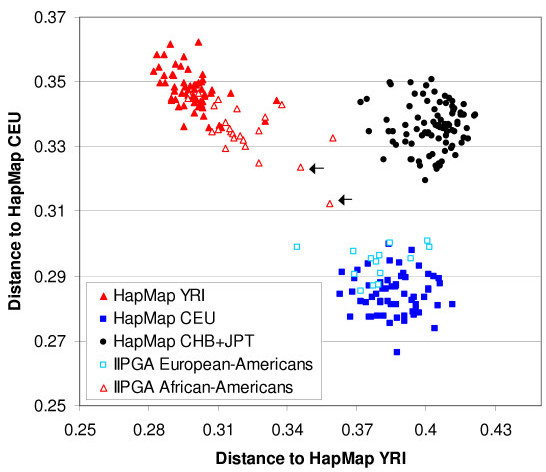
**Genetic distance from individuals in the IIPGA data to the three continentally defined HapMap groups**. Individuals cluster according to ancestral continent of origin but the clusters are less compact than those seen with the Perlegen data because of the smaller number of SNPs. Individuals intermediate between clusters, like those labeled with arrows, may have a high degree of admixture.

As an initial step in our investigation of genetic variation, for each individual we calculated the average genetic distance to the three continentally defined HapMap groups. We used an allele-sharing distance described by Mountain and Cavalli-Sforza as our measure of genetic distance [26]. Individuals clearly cluster according to ancestral continent of origin in both the Perlegen (Fig. 1) and IIPGA (Fig. 2) data sets. The clusters are most distinct in the Perlegen data set where the much larger number of SNPs provides greater resolution. The African-Americans in the test data sets cluster with the Yoruba, but the African-Americans are slightly closer to the European-American cluster. This is likely indicative of the fact that African-Americans represent a group of African origin that has had some degree of admixture with people of European origin. Finally, note that there are some points in the IIPGA data set (such as those labeled with arrows in Fig. 2) that lie between clusters. These individuals may have a particularly high degree of admixture.

**Figure 3:**
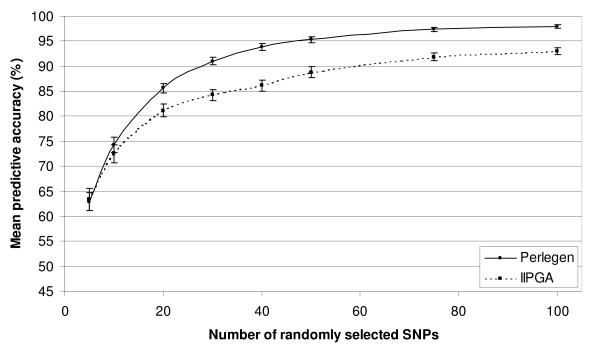
**Mean predictive accuracy in the test data sets**. Each data point represents the mean of 100 trials. Error bars depict 95% confidence intervals. Predictive accuracy increases with the number of SNPs used, but is still very good even with only a small number of SNPs.

The analysis described above shows that when large numbers of SNPs are used, it is readily apparent that genetic structure varies as a function of ancestral continent of origin. Next, in order to evaluate the extent of variation between continentally defined groups, we randomly selected varying numbers of SNPs and then used genotype information from the HapMap individuals to train a naïve Bayes classifier. Naïve Bayes is a simple predictive algorithm that has been shown perform well in a wide variety of situations [27]. We chose to use this algorithm because its simplicity and speed made it computationally feasible to test thousands of sets of randomly selected SNPs. Using naïve Bayes, we were able to accurately predict ancestral continent of origin with genotype information from only a small number of randomly selected SNPs (Fig. 3). For example, when only 50 randomly selected SNPs are used, mean predictive accuracy is 95% in the Perlegen data set and 89% in the IIPGA data set. Even with only 5 randomly selected SNPs, the observed predictive accuracies (63% in both the Perlegen and IIPGA data) are much higher than the 33% accuracy expected by chance.

**Figure 4:**
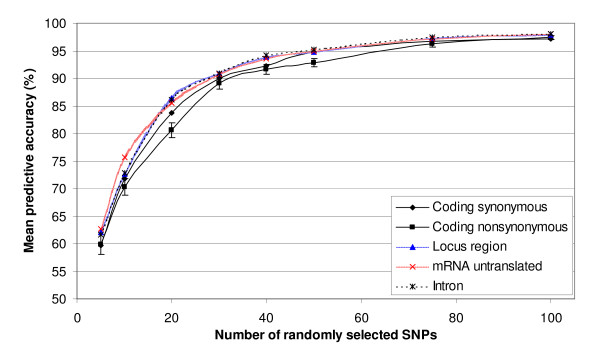
**Mean predictive accuracy stratified by type of SNP**. Each data point represents the mean of 100 trials. 95% confidence intervals are shown for the coding non-synonymous SNPs. Confidence interval widths are of comparable size for the other SNP categories. Predictive accuracy is very similar across all types of SNPs, although the small differences in mean predictive accuracy are statistically significant because of the large number of SNPs used in the analysis. For each n (where n is the number of randomly selected SNPs used to build the classifiers), one-way ANOVA was used to test the null hypothesis that means across SNP categories were equal. P-values were less than .01 for all tests where n>5, and p = .07 for n = 5.

The Perlegen data set is large enough to investigate genetic variation as a function of SNP type. We used the dbSNP database [28] to identify SNPs in introns, coding exons, regulatory regions and regions coding for untranslated mRNA. We then constructed naïve Bayes classifiers using only SNPs from a specific category. Predictive accuracies are very similar across all of the classifiers created in this manner (Fig. 4). However, more than one million SNPs were used in this analysis and the small differences in predictive accuracy across SNP categories were found to be statistically significant when compared using one-way ANOVA. The p-value was .0002 when we tested the null hypothesis that mean predictive accuracies were equal across SNP categories when 100 randomly selected SNPs were used to build classifiers. P-values were less than .01 for all tests where n>5 (where n is the number of randomly selected SNPs used to build classifiers). Predictive accuracy tended to be lowest when coding non-synonymous SNPs were used to build classifiers – although the absolute differences in predictive accuracy were extremely small.

**Figure 5:**
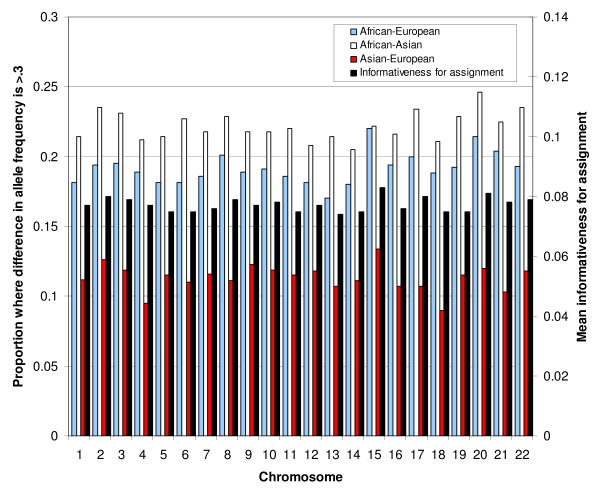
**Distribution of informative SNPs by chromosome**. The primary y-axis (on the left) shows the proportion of SNPs on each chromosome where the difference in major allele frequency between two of the continentally defined HapMap groups is greater than 0.3. Informativeness for assignment is shown on the secondary y-axis (on the right). The distribution of informative SNPs is fairly even across chromosomes. Since almost 4 million SNPs were used in this analysis, the relatively small observed differences are statistically significant however (p < .0001 for the difference in mean informativeness for assignment across chromosomes using one-way ANOVA).

**Figure 6:**
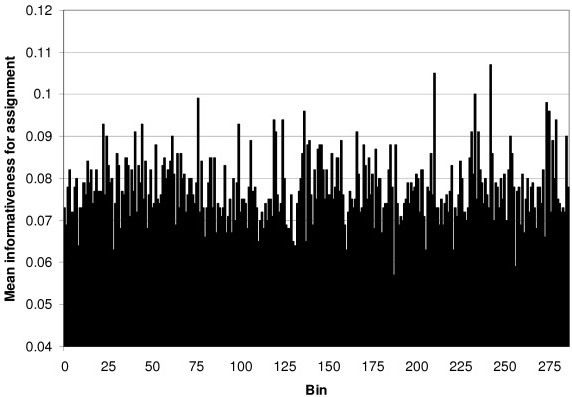
**Distribution of informative SNPs throughout the genome**. Each chromosome was divided into 10 M base pair bins. Bins near centromeres or near the ends of chromosomes containing only a very small number of SNPs were excluded. Bins contained an average of 3650 SNPs. The figure shows mean informativeness for assignment by bin for the entire genome. The mean informativenss for assignment is relatively similar over all the bins, although the small observed differences are statistically significant (p < .0001 with one-way ANOVA). Results were similar when 1 M base pair bins were evaluated.

We also tested whether some regions of the genome were more informative than others in predicting ancestral continent of origin. SNPs where allele frequency differs significantly between groups are most useful in predicting group membership. As one measure of informativeness, we evaluated pairwise difference in major allele frequency between groups. SNPs where the pairwise difference in major allele frequency was greater than .3 were considered to be informative. Other threshold frequencies were also evaluated and the relative distribution of informative SNPs was found to be similar across a range of thresholds. We also analyzed the informativeness for assignment. This measure was introduced by Rosenberg et al and is a generalization of the difference in major allele frequency to more than two groups [29]. The distribution of informative SNPs was relatively even throughout the genome (Figs. 5 and 6). One-way ANOVA was used to test whether the mean informativeness for assignment was equal throughout the genome. As was the case with SNP categories, the large number of SNPs analyzed resulted in the small observed differences being statistically significant (p < .0001 for tests of equality across both chromosomes and 10 M base pair bins).

## Discussion

In this paper, we use naïve Bayes classifiers trained on data from the HapMap to predict the ancestral geographic origin of individuals from three independent data sets. Even when a relatively small number of randomly selected SNPs are used, classification is accurate and robust. In the large Perlegen data set, predictive accuracy increases to 100% as the number of SNPs grows. This is not the case for the IIPGA test data set. Predictive accuracy as a function of the number of SNPs used, while still very good in comparison to many tests used in biology and medicine, levels off at 95% with two individuals being consistently misclassified. These two individuals were classified incorrectly even if all 1588 available SNPs were used to train the classifier. The two misclassifications were the two African-Americans labeled with arrows in Fig. 2 who were noted to be intermediate between the African and European clusters. These two individuals may have mixed African and European ancestry or may have African ancestors from different parts of Africa than the Yoruba used to train the classifier.

Accurate characterization of ancestry will allow investigators conducting genetic association studies to identify stratification in study subjects and avoid false positive genotype-phenotype associations. The analysis we describe here is designed to predict ancestral continent of origin. It could easily be extended to make predictions about smaller units of geography or individuals with a mixed background. This would require more extensive genotype data and well-characterized information about ancestral geographic origin from such individuals. There is only very limited data of this kind currently available, but this is expected to change in the future as genotyping costs decrease. Thus we anticipate that identification of more complex patterns of ancestry will be increasingly feasible as the amount of available data grows. This in turn will allow the development of higher resolution genographic maps and provide investigators designing genetic association studies with more powerful tools for detecting stratification.

In this paper, we have shown that the differences between continentally defined groups are sufficiently large that even a randomly selected, minute fraction of the genetic variation in the human genome can be used to characterize ancestral geographic origin in an accurate and reproducible manner. This argues strongly against the contention that differences between groups are too small to have biomedical significance. Understanding if and how these differences relate to risk of disease and response to therapy is one of the major challenges facing the biomedical research community.

## Conclusion

Some have argued that the differences between continentally defined groups are relatively small and that it is difficult to distinguish groups without using large amounts of genetic data or specifically chosen markers. Our results show that continentally defined groups can be easily distinguished using only a small number of randomly selected SNPs. SNPs that are informative about ancestry are common and widely distributed throughout the genome and across SNP types. These findings illustrate the extent of genetic variation between continentally defined groups.

## Methods

### Data sources

The HapMap, Perlegen and IIPGA data sets were obtained from their respective websites [25,30,31]. For the HapMap data we used the non-redundant data sets from public release 19 which contained data for phases I and II. Thirty trios were genotyped for both the Yoruba and European populations. We excluded the children from our analysis because they did not represent independent genotypes. We also excluded the one individual from the Japanese population who did not have phase I data. There were 60 Yoruba, 60 European-Americans, 45 Han Chinese and 44 Japanese. For the purpose of our analysis, the Han Chinese and Japanese subjects were grouped together.

For the IIPGA data set, we used dbSNP annotation files to map the IIPGA identifiers to the official NCBI reference SNP identifier [28]. The dbSNP annotation files were also used to determine SNP type.

Nine individuals in both the Perlegen and IIPGA data sets were also genotyped as part of the HapMap project. In our study, these individuals were included in the HapMap data set, but not in the Perlegen or IIPGA data sets.

### Genetic distance

The genetic distance between two individuals at a single loci was defined to be zero if the two individuals had the same genotype, 1/2 if they had one allele in common and 1 if they had neither allele in common (i.e. d(CG,CC) = .5 and d(CC,GG) = 1) [26]. The genetic distance between two individuals was calculated as the mean genetic distance over all loci genotyped in both individuals. The genetic distance between an individual and a group was defined as the mean of the pairwise genetic distances between the individual and all members of the group – except if the individual was a member of the group, we did not include the distance between the individual and him or herself. Genetic distances were normalized so that for each individual the genetic distances to the HapMap YRI, CEU and JPT+CHB summed to one.

### Classification with naïve Bayes

We wrote our own implementation of the naïve Bayes algorithm [32] in PERL. We assumed a uniform prior probability distribution over the class variable. For each test data set, we randomly selected a specified number of SNPs. We then used the HapMap data for these SNPs to train a classifier. The performance of this classifier was then evaluated by determining accuracy of prediction on the test data set. This process was repeated 100 times for the specified number of SNPs.

### Identification of SNPs informative about ancestry

To evaluate the distribution of SNPs where allele frequency differed significantly between groups, we computed the pairwise differences in major allele frequency among the three HapMap groups. For the purposes of our study, the most frequent allele in the Yoruba was considered to be the major allele. For each pairwise combination of groups, we determined the proportion of SNPs where the difference in major allele frequency exceeded a threshold value. We investigated how this proportion varied across the genome.

We also used the informativeness for assignment measure to analyze SNPs [29]. The informativeness for assignment (*I*) of a SNP is defined as:

I=∑j=1N(−pjlog⁡pj+∑i=1Kpijlog⁡pijK)
 MathType@MTEF@5@5@+=feaafiart1ev1aaatCvAUfKttLearuWrP9MDH5MBPbIqV92AaeXatLxBI9gBaebbnrfifHhDYfgasaacH8akY=wiFfYdH8Gipec8Eeeu0xXdbba9frFj0=OqFfea0dXdd9vqai=hGuQ8kuc9pgc9s8qqaq=dirpe0xb9q8qiLsFr0=vr0=vr0dc8meaabaqaciaacaGaaeqabaqabeGadaaakeaacqWGjbqscqGH9aqpdaaeWbqaaiabcIcaOiabgkHiTiabdchaWnaaBaaaleaacqWGQbGAaeqaaOGagiiBaWMaei4Ba8Maei4zaCMaemiCaa3aaSbaaSqaaiabdQgaQbqabaGccqGHRaWkdaaeWbqaamaalaaabaGaemiCaa3aaSbaaSqaaiabdMgaPjabdQgaQbqabaGccyGGSbaBcqGGVbWBcqGGNbWzcqWGWbaCdaWgaaWcbaGaemyAaKMaemOAaOgabeaaaOqaaiabdUealbaaaSqaaiabdMgaPjabg2da9iabigdaXaqaaiabdUealbqdcqGHris5aaWcbaGaemOAaOMaeyypa0JaeGymaedabaGaemOta4eaniabggHiLdGccqGGPaqkaaa@57D5@

where there are *N *alleles and *K *populations, *p*_*ij *_represents the frequency of allele *j *in population *i*, and *p*_*j *_represents the mean value of *p*_*ij *_over the *K *populations. This measure is a generalization of the absolute difference in allele frequency to more than two populations.

We analyzed the distribution of informative SNPs on both a chromosomal basis and by dividing the chromosomes into bins of a constant size. We tested multiple combinations of bin size and allele frequency cutoff threshold. The conclusion that SNPs useful for classification are distributed across the genome was not sensitive to changes in these parameters.

### Comparing means

One-way ANOVA (calculated using Microsoft Excel) was used to compare mean predictive accuracy across SNP types and to compare informativeness for assignment across both chromosomes and bins. The null hypothesis was that all means were equal. P-values less than .05 were considered to be statistically significant.

## Authors' contributions

DJA conducted the analyses and drafted the manuscript. QS and GG participated in the design of the study. MFR and ISK conceived of the study and participated in its design. All authors read and approved the final manuscript.
